# Single-Cell Transcriptome and Network Analyses Unveil Key Transcription Factors Regulating Mesophyll Cell Development in Maize

**DOI:** 10.3390/genes13020374

**Published:** 2022-02-20

**Authors:** Shentong Tao, Peng Liu, Yining Shi, Yilong Feng, Jingjing Gao, Lifen Chen, Aicen Zhang, Xuejiao Cheng, Hairong Wei, Tao Zhang, Wenli Zhang

**Affiliations:** 1 State Key Laboratory for Crop Genetics and Germplasm Enhancement, Collaborative Innovation Center for Modern Crop Production Co-Sponsored by Province and Ministry (CIC-MCP), Nanjing Agricultural University, No.1 Weigang, Nanjing 210095, China; 2019101149@njau.edu.cn (S.T.); 2020101155@stu.njau.edu.cn (Y.S.); 2017201057@njau.edu.cn (Y.F.); 2020101156@stu.njau.edu.cn (J.G.); 2016101164@njau.edu.cn (L.C.); 2018201003@stu.njau.edu.cn (A.Z.); xuejiaocheng@njau.edu.cn (X.C.); 2 Joint International Research Laboratory of Agriculture and Agri-Product Safety of the Ministry of Education, Yangzhou University, Yangzhou 225009, China; pengliu@yzu.edu.cn; 3 College of Forest Resources and Environmental Science, Michigan Technological University, Houghton, MI 49931, USA; hairong@mtu.edu; 4 Jiangsu Key Laboratory of Crop Genomics and Molecular Breeding, Key Laboratory of Plant Functional Genomics of the Ministry of Education, Agricultural College of Yangzhou University, Yangzhou 225009, China

**Keywords:** scRNA-seq, M-cell development, pseudo-time analysis, coexpression and regulatory network, transcript factor, maize

## Abstract

Background: Maize mesophyll (M) cells play important roles in various biological processes such as photosynthesis II and secondary metabolism. Functional differentiation occurs during M-cell development, but the underlying mechanisms for regulating M-cell development are largely unknown. Results: We conducted single-cell RNA sequencing (scRNA-seq) to profile transcripts in maize leaves. We then identified coregulated modules by analyzing the resulting pseudo-time-series data through gene regulatory network analyses. *WRKY*, *ERF*, *NAC*, *MYB* and *Heat stress transcription factor* (*HSF*) families were highly expressed in the early stage, whereas *CONSTANS (CO)-like* (*COL*) and *ERF* families were highly expressed in the late stage of M-cell development. Construction of regulatory networks revealed that these transcript factor (TF) families, especially *HSF* and *COL*, were the major players in the early and later stages of M-cell development, respectively. Integration of scRNA expression matrix with TF ChIP-seq and Hi-C further revealed regulatory interactions between these TFs and their targets. *HSF1* and *COL8* were primarily expressed in the leaf bases and tips, respectively, and their targets were validated with protoplast-based ChIP-qPCR, with the binding sites of HSF1 being experimentally confirmed. Conclusions: Our study provides evidence that several TF families, with the involvement of epigenetic regulation, play vital roles in the regulation of M-cell development in maize.

## 1. Introduction

As a C4 plant species, maize plays an increasingly important role in global grain production. Its high yield potential is rooted in its leaf structure and functional differentiation of mesophyll (M) and bundle sheath (BS) cells. Maize leaves form a classical Kranz leaf anatomy, which is featured by closely spaced parallel veins that are encompassed by two morphologically distinct photosynthetic cell types: a ring of BS cells followed by one or more concentric files of M cells. BS cells are characterized by thick cell walls and centrifugally arranged chloroplasts with large starch granules and unstacked thylakoid membranes, whereas M cells are generally characterized by randomly arranged chloroplasts with stacked thylakoids and little or even no starch [1]. The anatomically distinct chloroplasts in M and BS cells give them some distinct functions. For example, chloroplasts in M cells contain grana thylakoids where both PSII and PSI systems perform linear electron transport, and photoreduction of NADP^+^ for fixing CO_2_. In contrast, BS cells have agranal chloroplasts and lower PSII activity, and perform most of the reactions of the Calvin cycle [2]. In addition, biochemical studies have revealed that the enzymes involved in lipid biosynthesis, nitrogen import, and tetrapyrrole and isoprenoid biosynthesis are preferentially located in M chloroplasts, while the enzymes involved in starch synthesis and sulfur import preferentially accumulate in BS chloroplasts. The coordination of anatomical and biochemical components makes the C4 plant species possess the most efficient photosynthetic carbon assimilation system. To advance our understanding of highly photosynthetic capacity for carbon assimilation in C4 plants, it is important to understand the regulatory mechanisms underlying functional differentiation of M and BS cells.

The underlying mechanisms controlling functional differentiation between maize M and BS cells have been intensively studied at the levels of biochemical rationales [3,4,5], gene expression [6,7,8] and gene regulation [9,10,11,12,13,14]. Different soluble antioxidative compounds, for example peroxiredoxins that can mitigate photo-oxidative stress and damage, have been found to be accumulated at higher levels in the chloroplasts of mesophyll cells [4]. Several genes, such as *PPDK*, *psa1* and *psa2*, with functions in chloroplast biogenesis, have been genetically identified and experimentally verified in maize [15], though their roles in regulating the M-cell development are largely unknown. These questions are difficult to answer using the conventional bulk M-cell RNA-seq approach that neutralizes the expression level of each gene from different M cells.

Here, we studied M-cell development and regulation by conducting a single maize-leaf-cell RNA sequencing (scRNA-seq) followed by pseudo-time data analyses. We revealed that *WRKY*, *ERF*, *COL*, *NAC*, *MYB* and *HSF* transcription factor (TF) families were involved in functional differentiation of M cells during their development. Especially, *COL* and *HSF* families functioned in the early and later stage of M-cell development, respectively. We found that *HSF1* and *COL8* (a mesophyll cell-development regulator) functioned in base and tip development, respectively, and some genes were coregulated by HSF1 and COL8 in the base but not regulated by COL8 in the tip. In addition, the binding sites of HSF1 were experimentally confirmed in maize. Thus, our study provides evidence showing that M-cell development is regulated through a TF-related regulatory network in maize.

## 2. Materials and Methods

### 2.1. Plant Growth

Maize (*Zea mays* “B73”) seeds were germinated at room temperature (RT). The germinated seeds were transferred to pots with soil and grown in a growth chamber with a temperature between 25 °C and 28 °C and a 16 h/8 h light–dark cycle. The plants were grown for 10 days for leaf collection. The collected 10-day-old leaves were used for preparation of mesophyll (M) and bundle sheath (BS) cells for single-cell (scRNA-seq) or bulk RNA-seq experiments.

### 2.2. Preparation of M and BS Cells

Isolation of M or BS cells was conducted according to the published procedures with some minor modifications [7]. Briefly, the intact 10-day-old second leaf from individual trifolium-stage plant was cut into slices with the long axis to 0.5–1 mm using a single-edge razor blade.

Isolation of M cells: The leaf slices were immediately placed into a Petri dish containing an enzymatic digestive medium (0.6 M D-Mannitol, 1.5% (*w*/*v*) cellulose (Onozuka RS), 0.75% (*w*/*v*) macerozyme (Onozuka R-10), 0.1% BSA, 20 mM KCl, 10 mM CaCl_2_ and 10 mM MES, pH 5.8), and vacuumed for 30–60 min. The digestion was completed under dark condition on a shaker with speed of 55 rpm at 26 °C for 4 h. The digestive solution was diluted to release M cells, as many as possible, from the leaf slices, by adding 10 mL W5 buffer (154 mM NaCl, 125 mM CaCl_2_, 5 mM KCl, 5 mM glucose and 0.03% MES, pH 5.8). We then used a 35 µm nylon net to separate M cells from BS cells. After filtration, the filtrate was pelleted by a centrifuge at 80× *g* for 5 min, and the pellet was then washed in the W5 buffer 3–4 times. The purity of M cells was checked under a bright-field microscope. The purified M-cell pellet was quick-frozen in liquid nitrogen and stored at −80 °C for later use. The isolated M cells were used for one replicate of scRNA-seq experiment using 10× Genomics Chromium and the two biological replicates of bulk RNA-seq.

Mechanical isolation of BS cells: The leaf slices were immediately placed into a blender containing 50 mL pre-cold BS cell-extraction buffer (0.6 M Sorbitol, 50 mM Tris-HCl pH 8.5, 5 mM EDTA, 0.5% PVP-10 and 0.01 M DTT). The leaf slices were homogenized three times, each for 10 s with a high-speed setting. The homogenized mixture was sequentially filtered through a 500 µm and then an 80 µm nylon mesh. BS cells retained on the mesh were then transferred to the 50 mL corning tube using 10 mL W5 buffer. The pellet was washed in the W5 buffer 3–4 times by repeating the above procedures of centrifuge (300× *g* for 5 min) and resuspension. The purity of BS cells was examined under a bright-field microscope. The purified BS cell pellet was quick-frozen in liquid nitrogen and stored at −80 °C until they were used. Two biological replicates of BS cells were used for bulk RNA-seq.

### 2.3. Reverse Transcription and Quantitative RT-PCR (qRT-PCR) Assay

Total RNA was extracted from the base and the tip samples of 10-day-old maize leaves using TRIzol (Invitrogen, Waltham, MA, USA). After completely removing genomic DNA contamination using DNase I, DNA-free RNA was used for synthesis of the first-strand cDNA using the 5ÍhiScript II qRT SuperMix II (Vazyme, R223-01-AC, Nanjing, China). qRT-PCR was performed according to the published procedures [16] with the primers being listed in Supplementary Table S1.

### 2.4. ChIP-qPCR Assays of Genes Regulated by the HSF1 and COL8 Transcription Factors

Genomic DNA sequences of the *heat shock factor1* (*HSF1*) and *CONSTANS-like 8* (*COL8*) gene were inserted into pCAMBIA 1305 vector, where *HSF1/COL8* fused with a GFP tag under the control of CAMV 35S promoter. The tip and the base part of the second leaf collected from 10-day-old maize plants with three-leaf stage was used for M-cell preparation following the aforementioned procedures. The plasmid DNA vectors harboring *HSF1* and *COL8* genes fused with GFP were transiently expressed in the M cells generated from the leaf bases and tips, respectively, using PEG-mediated transfection. Upon 24 h incubation under dark condition at 26 °C, the transfected M cells were cross-linked using 1% formaldehyde and collected for anti-GFP antibody (ab290, abcam) ChIP assay following the published procedures [16]. Biologically replicated ChIP-qPCR was performed according to the published procedures [16] with the primers being listed in Supplementary Table S1.

### 2.5. Validation of the HSF1-Binding Motif

The HSF1-binding motif (AGAAnnTTCT) in maize was validated using HSF1-IP-PCR assay and dual-luciferase reporter gene assay.

For HSF1-IP-PCR assay, *HSF1* overexpression plasmid DNA and the plasmid containing promoter of *NAC75* (*P_NAC75_*) with the HSF1-binding and corresponding scrambled sequences were cotransformed into M cells using the same procedures as for the aforementioned HSF1-ChIP-qPCR assay. Anti-GFP antibody was used to pull down the GFP fused with HSF1. The resulting DNA was used for PCR assays with the primers listed in Supplementary Table S1.

For dual-luciferase reporter gene assay, the coding sequences of *HSF1* gene were used for constructing the expression cassette containing 35S::*HSF1*; the promoter sequences and the corresponding scrambled sequences of *NAC75* were used for generating the expression cassettes of *P_NAC75_*: *LUC*. The agrobacteria containing each reporter gene were coinfiltrated into *Nicotiana benthamiana* leaves with combination of *P_NAC75_*: LUC with 35S::*HSF1*. LUC signal was recorded using a chemiluminescence imaging system named Tanon-5200 (Tanon Science).

### 2.6. Bulk RNA-Seq and Data Analyses

Bulk RNA-seq was carried out as previously described [17]. Bulk RNA-seq data for BS and M cells were aligned to the *Zea mays* AGPv4 reference genome using HISAT2 v2.1.0 [18]. The maize ensemble version of B73v4.43 gene annotation was used for calculating gene expression levels using StringTie 1.3.3b [19]. DESeq2 [20] was used to calculate differential gene expression (fold change > 2, q < 0.01). The expected gene numbers for each transcript factor (TF) family in the highly expressed genes were calculated using the highly expressed TF numbers multiplied by the ratios of TF numbers accounting for all TFs in each family in BS and M cells, respectively. Differentially enriched TF families between BS and M cells beyond the cutoff threshold (*p*-value < 0.01, chi-squared test) were also detected.

### 2.7. ChIP-Seq and MH-Seq Data Analyses

ChIP for H3K27me3, H3K4me3 and H3K36me3 histone marks in M cells and MNase hypersensitivity (MH) of whole-leaf assays and sequencing library construction were performed following the published protocols [17,21]. All ChIP-seq and MH-seq libraries were sequenced on the Illumina Hiseq 4000 platform. The published ChIP-seq data of the H3K27ac mark (PRJNA391551) were obtained from Sequence Read Archive (SRA) and the reads were trimmed using fastp (v0.19.4) [22]. Clean reads for ChIP-seq and MH-seq were aligned to *Zea mays* AGPv4 reference genome using BOWTIE2 v2.3.2 [23]. The regions from the upstream 1.5 kb to the downstream 1.5kb of TSSs were divided into bins with a size of 100 bp window, and the mean values of normalized read counts were calculated for each bin. MH-seq peak calling was performed using MACS2 [24] with parameters as -f BAMPE g 2.2e9 --nomodel -q 0.001 --fe-cutoff 5.

### 2.8. Single-Cell RNA-Seq (scRNA-Seq)

The maize leaf single cells (protoplasts) were prepared following exactly the same procedures as for isolation of bulk M cells mentioned above. All isolated individual cells were subjected to the quality control, which included the examination of cell integrity under bright field microscope, and the staining of cells with Trypan blue for testing cell viability. Cells were counted using the Countess II Automated Cell Counters (Thermo Fisher Scientific, Waltham, MA, USA) and adjusted to a concentration of 100–500 cells/µL. Actually, the viable cell concentration was about 300–1500 cells/µL due to underestimation of viable cell numbers by Cell Counters, which was caused by dark staining of some viable green M cells with Trypan blue staining. The cell counter-related underestimation of viable green protoplasts with Trypan blue staining can be improved by using hematocytometer to count cells manually under the microscope. Single intact cells with over 80% viability were used to index single cells for 10× Single-Cell RNA-seq (scRNA-seq) library preparation using the 10× scRNA-seq platform (10× Genomics, Pleasanton, CA, USA) following the manufacture’s instruction. The barcoded scRNA-seq libraries were finally sequenced using the Illumina NovaSeq platform. Both scRNA-seq library construction and sequencing were performed by Berry Genomics (Beijing, China).

### 2.9. Preprocess of scRNA-Seq Data

The raw scRNA-seq data were aligned to the AGPv4 reference genome by Cell Ranger (v3.0.2). The files in the dir filtered_feature_bc_matrix were imported into R for constructing the expression matrix using the Seurat packages [25]. Cells with read numbers less than 10,000 and mitochondria ratio higher than 0.2 were considered as low-quality and were filtered out. The FindVariableFeatures function in Seurat was employed to calculate the average expression level and dispersion for each gene. The top 1500 dispersed genes were used to perform PCA using the RunPCA function. To reduce the dimension, we chose UMAP (Uniform Manifold Approximation and Projection), which has a similar visualization quality to the tSNE (t-Distributed Stochastic Neighbor Embedding) but may preserve more of the global structure [26]. Through UMAP analyses, the dimension of the first 25 principal components was reduced into 2 components using the RunUMAP function with the parameters n.neighbors = 50 and min.dist = 0.1. All cells were clustered based on the 2 UMAP dimensions with Louvain methods using the FindNeighbors function with dims = 1:50 and the FindClusters function with resolution = 0.05. The published maize M-cell-related scRNA-seq data (GSE157759) were preprocessed using the same workflow, but the cutoff value related to the read number of cells were set to 3000.

### 2.10. Pseudo-Time Analyses

The cell-dataset (CDS) was generated from the M-cell Seurat object by using ‘as.CellDataSet’ functions in Seurat. Pseudo-time analyses were then performed with the dataset in the CDS format using the Monocle 2 package [27]. The cell data sets were preprocessed using ‘estimateSizeFactors’ and ‘estimateDispersions’ functions. Dispersion table was calculated and genes with mean expression levels > 0.1 were considered as expressed genes for the downstream analyses. The differentially expressed genes (DEGs) were detected using ‘differentialGeneTest’ and the genes with vst.variable = True were then used to perform dimension reduction using reduceDimension function with parameters set as “max_components = 2, method = ’DDRTree’’. Cells were ordered according to the pseudo-time values by the function orderCells.

### 2.11. Motif Analyses

Transcription factor (TF)-binding motifs for WRKY, ERF, NAC, MYB and HSF families were downloaded from C3C4 database (http://www.epigenome.cuhk.edu.hk/C3C4.html, accessed on 3 January 2022). Enrichment analyses of the TF binding sites within MH peaks were performed using Analysis of Motif Enrichment (AME) in the MEME motif enrichment function.

### 2.12. Weighted Coexpression Network Analysis (WGCNA)

Cells were divided into 22 clusters using ‘FindClusters’ with the parameter resolution being set to 0.5. RNA-seq reads from the cells in each cluster were pooled and considered as an M-cell sample with pseudo-time, which was calculated as the mean value for all cells in the same cluster. The expression levels were normalized to log_2_^(RPM values)^. The top 75% dispersed genes were used to construct coexpression connections. Coexpressed analysis was performed using the WGCNA package [28].

### 2.13. Construction of TF Regulatory Network Using SCODE

The pseudo-time for each cell was normalized from 0 to 1. The expression matrix for TFs was extracted from Monocle CellDataSet. Gene regulatory network associated with dynamic TFs was constructed using the SCODE [29] with the parameter z being set to 4. Average results of 50 runs were used to acquire reliable connections between TFs. The network was analyzed and visualized using the Cytoscape [30].

### 2.14. Construction of Gene Regulatory Network

Edges between the transcription factors and target genes were constructed with the published ChIP-seq data of 104 transcription factors (PRJNA518749), which were also generated from maize bulk M-cell-related protoplasts [31]. PPI (promoter–promoter interaction) was constructed when two genes showed spatial interactions through consecutive H3K4me3 HiChIP loops. PDI (promoter–distal interaction) was constructed based on the published dACR-gene interaction loops and ChIP-seq peaks of transcription factors when the peaks of transcription factors overlap dACRs in the loops [32].

### 2.15. GO-Term Enrichment Analyses

GO enrichment analyses were performed using the online tools in plant regulomes database (http://bioinfo.sibs.ac.cn/plant-regulomics, accessed on 3 January 2022) [33] with *Zea mays* annotations. The redundant GO-term enrichments were removed using online tools in REVIGO (http://www.incodom.kr/REVIGO, accessed on 3 January 2022) [34].

### 2.16. Visualization of H3K4me3 HiChIP Network

The H3K4me3 HiChIP loop tracks were downloaded from the published epi-genome browser (http://epigenome.genetics.uga.edu/PlantEpigenome/, accessed on 3 January 2022) [32]. The Cytoscape was used to visualize the network [30].

### 2.17. General Bioinformatics Software

The Sra Toolkit was used to download next-generation sequencing data in the sra format (.sra) from the Sequence Read Archive (SRA) database. Data were converted to the compressed fastq format using the fastq-dump function in the Sra Toolkit. FastQC was used to perform quality control for the ChIP-seq and RNA-seq dataset. Deeptools was used to generate tracks in BigWig format (.bw) for ChIP-seq dataset. Samtools was used to process alignment data in bam format. The Bedtools was used to analyze genomic coordinate datasets such as peak files and gene annotation files. R was used to perform some data analyses and for plotting the figures.

## 3. Results

### 3.1. Single-Cell RNA (scRNA) Sequencing of the Maize Leaf

**Figure 1:**
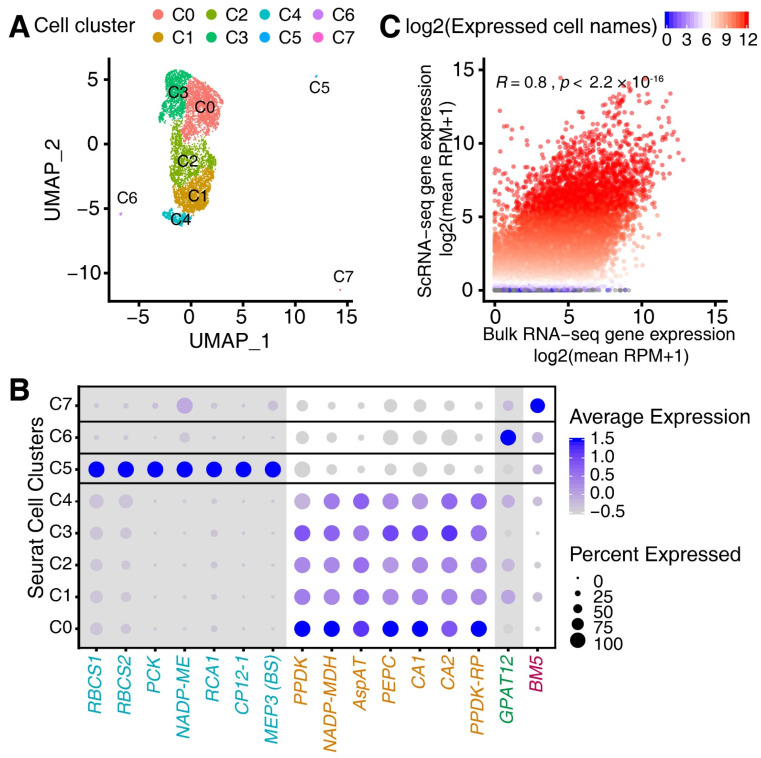
Characterization of scRNA-seq. (**A**) UMAP-based scatterplot showing eight cell clusters for 7354 filtered cells. (**B**) Dotplot showing the average expression levels for 16 cell-type specific genes differentially expressed between M and BS cells and other cell types in the 8 Seurat clusters. The size of the dot represents the ratio of cells with expressed genes out of all cells. Genes with higher expression levels in the BS cells were displayed on a grey background. (**C**) Scatter plot showing the log_2_^(mean RPM+1)^ for all genes in the bulk RNA-seq data (*x* axis) and scRNA-seq data (*y* axis). The color of each dot represents the log_2_^(cell numbers with expressed genes in the scRNA-seq)^. The correlation test was determined using Spearman correlation test.

We conducted single maize leaf-cell RNA sequencing (scRNA-seq) and obtained 656,666,388 scRNA-seq reads (Q30 base content: 93.1% for RNA reads, 95.7% for Barcode and 95.1 for UMI). The estimated cell numbers were 14,656 with a mean of read numbers equal to 44,805 per cell and 93.7% of them were mapped to the B73 reference genome (v4, gene annotation ensemble 4.43) (Supplementary Dataset S1). Initially, we obtained approximately 14,656 cells, 44,805 reads per cell, 2148 median genes, and detected more than 28,765 total genes in the population. After quality control (see Section 2), we further filtered out the cells with the low number of reads and the reads originating from mitochondrial genes. We finally obtained 7354 cells with more clean reads and few mitochondria contaminations for analyses. We then used UMAP (Uniform Manifold Approximation and Projection) to reduce the dimension and obtained eight major supervised cell clusters (C0–C7, C is short for Cell) using a parameter setting of ‘n.neighbors = 50’ and ‘min.dist = 0.1’ (Figure 1A). Moreover, by using the average expression levels of several well-characterized marker genes [7] between BS and M cells in maize (Figure 1B), we revealed that Cluster C0–C4 (*n* = 2192, 1695, 1527, 1375 and 465, respectively) were M-cell clusters, whereas Cluster C5 was BS-cell clusters (*n* = 38). To compare the homogeneity of our scRNA-seq and the newly generated bulk RNA-seq using the plants grown in the same condition, the Spearman rank correlation was applied to the mean RPM (Reads per million mapped reads) values for M-cell bulk RNA-seq and the expression matrix of M cells from scRNA-seq data, with all values being prenormalized before a log_2_ transformation. We found that gene expression profiles between scRNA-seq and conventional bulk M-cell RNA-seq data were highly correlated (Spearman, *R* = 0.87, *p* < 2.2 × 10^−16^) (Figure 1C). Moreover, we also found that our scRNA-seq data exhibited a high correlation with the recently published scRNA-seq data generated from M cells of maize seedlings that were preprocessed in the same way [35] (Supplementary Figure S1). In addition, we found that epidermis-related marker gene *GPAT12* was highly expressed in Cluster C6 (*n* = 34) and vascular sclerenchyma-related marker gene *BM5* was highly expressed in C7 (*n* = 28), suggesting the possible origin of both cell clusters. Since these two cell clusters showed large differences in the UMAP component 1, it is also possible that they belong to variant M/BS cell types or are likely a consequence of contamination of other specialized cell types during M/BS cell preparation. Specifically expressed genes for Cluster C6 and C7 were identified using Seurat and are listed in Supplementary Table S2. Gene Ontology (GO) term enrichment analysis of these genes showed that functions of specific genes in Cluster C6 were mainly involved in the (sub) cellular membrane and transferase or catalytic activity, and the genes in Cluster C7 were overrepresented in the cellular site of cytoplasm and a function of nucleotide binding (Supplementary Figure S2). These results showed that the scRNA-seq data we generated can reveal the expression levels of maize leaf single cells, thus can be used for the downstream analyses, and analytic procedures and methods we performed were sufficient in generating cell transcriptomes representing major cell tissue types. Thus, we ultimately decided to use 7254 M cells for further characterization of M-cell development.

### 3.2. Characterization of Developmental Stage of M Cells Using Pseudo-Time Trajectory Analyses

**Figure 2:**
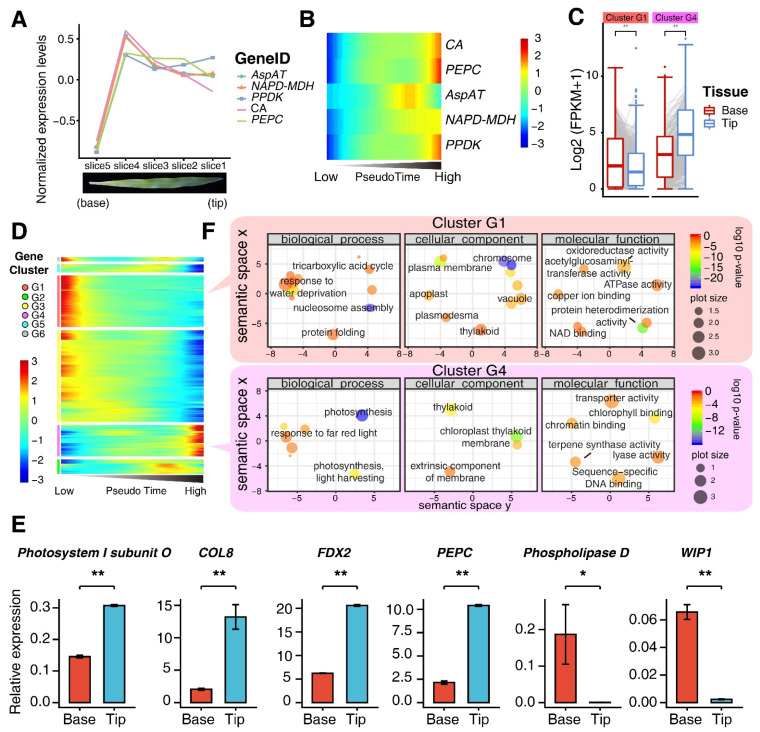
Dynamic expression profiles across pseudo time. (**A**) Expression profiles for *AspAT*, *NAPD-MDH*, *PPDK*, *CA* and *PEPC* genes in the bulk M-cell RNA-seq data generated from different dissected parts of the single leaf starting from the base to the tip as shown in the diagram (top) and the image form the real leaf (bottom). (**B**) Heatmap showing expression levels of *AspAT*, *NAPD-MDH*, *PPDK*, *CA* and *PEPC* across the pseudo-time. (**C**) Expression levels for genes in Cluster G1 and Cluster G4 in the base and the tip from the published data sets. Boxplot showing expression levels for genes in Cluster G1 and Cluster G4 in the base and the tip from the published data sets. Significance test was determined using two-tailed Wilcoxon method (** indicates *p* < 0.01). (**D**) Expression heatmap exhibiting 2584 highly dynamically expressed genes across the pseudo-time during M-cell development. (**E**) RT-qPCR assays using genes selected from Cluster G1 and G3 and G4, respectively, which were conducted using mRNA extracted from the leaf bases and the leaf tips, respectively. The relative expression levels were presented as mean ±SD from three technical replicates. Significance test was determined using one-sided *t*-test, * indicates *p* < 0.05, ** indicates *p* < 0.01. (**F**) Semantic similarity-based scatterplot showing the enriched GO terms for Cluster G1 and Cluster G4 genes. The color of each dot represents the log_10_^(*p*-value)^ for the significance of the GO-term enrichment; the size of each dot represents the frequency of the GO term in the underlying GOA database (more general terms exhibit larger bubbles).

To assess development stage of individual single M cells, we conducted the pseudo-time trajectory analyses using the Monocle 2 software package [27]. In brief, we specifically annotated each single cell with a pseudo-time value, which was calculated based on the expression matrix of genes within 7254 single M cells. We then obtained pseudo-time-based expression profiles of all genes from all cells (see methods). Generally, lower pseudo-time values represent earlier developmental stages while higher pseudo-time values reflect later developmental stages. The primary origin of the trajectory was determined by the five marker genes, *Aspartate transaminase* (*AspAT*), *NADP-malate dehydrogenase* (*NADP-MDH*), *pyruvate, Pi dikinase* (*PPDK*), *carbonic anhydrase* (*CA*), and *phosphoenolpyruvate carboxylase* (*PEPC*), as shown in Figure 2A,B. It could be switched between the other ends of the trajectory (Supplementary Figure S3). The origin of the trajectory was ultimately determined based on some biological facts, especially the M-cell marker genes that are provided below.

To determine the origin of cell state of the pseudo-time analysis, we analyzed expression profiles of five M-cell-specific genes, including *AspAT*, *NADP-MDH*, *PPDK*, *CA*, and *PEPC* which are primarily involved in photosynthesis [9]. Those genes have been reported to show the lowest expression levels in leaf base [9]. It has been reported that the leaf base corresponds to the young cell-development stage, while the leaf tip reflects the old and mature cell stage [36]. We further profiled the expression levels of these five genes in different sections of a single maize leaf using the published bulk M-cell-related RNA-seq data, which were generated from five different sliced pieces of a single maize leaf starting from the base to the tip [37]. We found that all of them manifested much lower expression levels in the base slice compared with the rest of slices, indicating that the spatially differential expression levels of these genes align well with the trend of M-cell maturation (Figure 2A). Based on the expression profiles of the five genes, the origin was adjusted to match the expression trend as shown in the previously published data. Finally, we constructed a pseudo-time heatmap where these five genes were lowly expressed at the low end of pseudo-time (left) but were highly expressed at the high end of pseudo-time (right) (Figure 2B). As a result, the origin of the trajectory was determined based on the expression profiles of the five M-cell-specifically expressed genes.

Transgenic studies of genes with loss of function or overexpression showed that 67 genes play vital roles in two distinct stages, proliferation and expansion, of leaf development in various species [38]. They showed that 29 out of 67 genes decrease while 38 out of 67 genes increase during maize leaf development processes [38]. We then analyzed these genes in our scRNA-seq and found that 24 out of 29 genes decreased while 32 out of the 38 genes increased throughout the pseudo-time expression profiles, indicating the validity of the origin for generating the pseudo-time trajectory analysis (Supplementary Table S3).

2584 pseudo-time-related differentially expressed genes (DEGs) (see methods for identification of DEGs) were further divided into six gene clusters, referred to as Cluster G1-G6, according to differential profiles of gene expression (Figure 2C). The published transcriptomic datasets of maize leaves from base to tip were also analyzed [6]. We found that genes in G1 and G4 displayed inverse expression profiles. Genes in G1 and G4 were highly expressed in the low and high pseudo-time, corresponding to the early (base) and the late (tip) developmental stages of leaf tissues, respectively (Figure 2D). Pseudo-time analyses were also performed using the public repository scRNA-seq data (GSE157759) [35], we obtained 651 overlapping genes showing the same expression profile as genes in G1 and G3, which were illustrated in Figure 2C, and 149 overlapping genes showing the same expression profile as genes in G4, which were illustrated in Figure 2C between two datasets. (Supplementary Figure S4A). Furthermore, several genes were selected for qRT-PCR assays, which confirmed that *COL8*, *FDX2*, *PEPC*, *Photosystem I subunit O* were highly expressed in the tip and *WIP1* and *Phospholipase D* were highly expressed in the base. (Figure 2E). GO-term enrichment analyses using genes in G1 and G4, which had inverse pseudo-time expression profiles, revealed distinctly prominent biological potential functions (Figure 2F). Genes in G1 were mainly involved in biological processes such as nucleosome assembly, tricarboxylic acid (TCA) cycle, and response to water deprivation, whereas genes in G4 were involved in photosynthesis, providing additional evidence that supports the accuracy of the designated trajectory origin in the pseudo-time.

Collectively, these analyses indicate that pseudo-time trajectory analysis helps to dissect transcriptomic heterogeneity in complex tissues and study transcriptomes of numerous cell types simultaneously.

### 3.3. Epigenomic Signatures of Genes in G1 and G4

**Figure 3:**
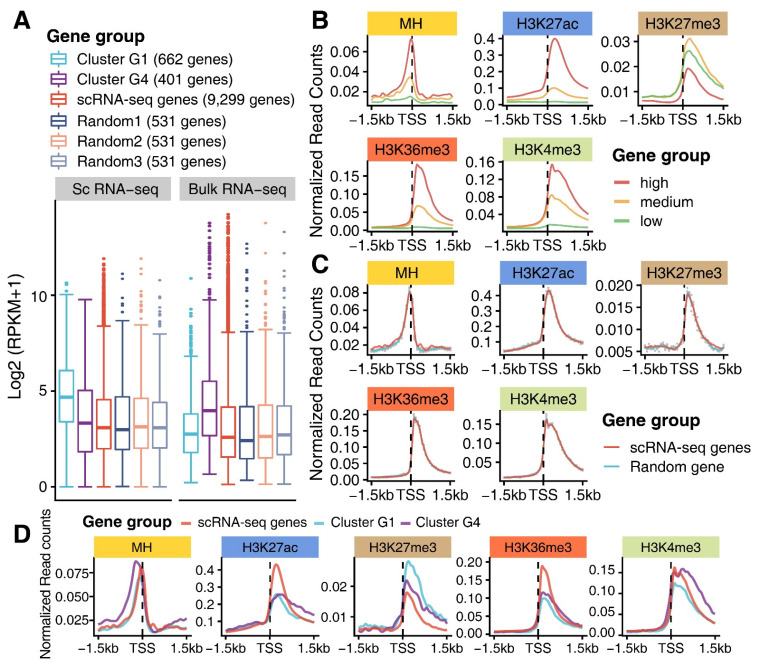
Characterization of expressed genes in the scRNA-seq data. (**A**) The expression levels (RPKM) of genes in different gene groups between the scRNA-seq and the bulk RNA-seq data sets. Random gene numbers of all genes were determined using the mean value of the gene numbers between Cluster G1 and Cluster G4 genes. (**B**) Normalized read counts for MHS and several histone marks across ±1.5 kb regions of TSSs of all annotated genes with different expression levels (RPKM, high, medium and low, each with the same number of genes) in the maize genome. Active marks (MHS, H3K27ac, H3K36me3 and H3K4me3) display a positive correlation with the expression levels of overlapping genes, while repressive mark, H3K27me3, is overall anticorrelated with the expression levels of overlapping genes. (**C**) Normalized read counts for MHS and several histone marks (H3K27ac, H3K36me3, H3K4me3 and H3K37me3) across ±1.5 kb regions of TSSs for the scRNA-seq genes and the mean values for three random replicates, the grey point represents actual values for the three replicates. (**D**) Normalized read counts for MHS and several histone marks (H3K27ac, H3K27me3, H3K36me3 and H3K4me3) across ±1.5 kb regions of TSSs for genes in Cluster G1 and Cluster G4, the scRNA-seq genes were used as control.

After scrutinizing the gene expression levels of several clusters, we found that the expression levels of 25% quantile, median, and 75% quantile of the genes in G1 were higher than those of the genes in G4 in the scRNAs-seq data, but were less than those of genes in the G4 in the bulk RNA-seq (Figure 3A). To explore whether distinct epigenomic regulations exist across different genes, we scrutinized epigenetic signatures upon integrating MNase hypersensitive sequencing (MH-seq), which was generated from the 10-day-old maize leaves, with our newly generated (H3K27me3, H3K4me3 and H3K36me3) or published (H3K27ac) ChIP-seq data [39] that were generated from the bulk M cells. After dividing all annotated maize genes into low, medium and high expression levels (FPKM values), we plotted the normalized read counts from each epigenetic mark spanning ±1.5 kb of the transcription start sites (TSSs) of all annotated genes. We observed that the active marks, MNase hypersensitive sites (MHSs), H3K36me3, H3K4me3 and H3K27ac, were positively correlated with gene expression levels, whereas the repressive mark, H3K27me3, was largely anticorrelated with gene expression levels (Figure 3B). The mean values for three random scRNA-seq gene sets with the equivalent numbers of genes (each containing 531 genes) to those in G1 (662 genes) and G4 (401 genes) displayed similar trends as all scRNA-seq genes, indicating that the number of genes did not affect the overall enrichment levels of histone marks examined (Figure 3C). We then performed similar analyses to in Figure 3B for all scRNA-seq genes (genes identified from the scRNA-seq data), G1 and G4 scRNA-seq genes. We observed that the scRNA-seq genes in G4 were more enriched with active marks, but less enriched with H3K27me3 compared with the scRNA-seq genes in G1, whose expression levels of 25% quantile, median and 75% quantile were higher than those of genes in G4 (Figure 3A,D). Compared with the scRNA-seq genes in G1 and G4, scRNA-seq genes with the lowest median expression levels in the scRNA-seq and the bulk RNA-seq manifested diverse enrichment profiles of these marks (Figure 3D). However, it is still explicit that scRNA-seq genes had the highest enrichment of H3K27ac and H3K36me3, and the lowest enrichment of H3K27me3 (Figure 3D). Unlike what we detected in Figure 3B, active marks and H3K27me3 did not exhibit a positive and negative correlation with gene expression levels of three types of scRNA-seq genes (all scRNA-seq genes, scRNA-seq genes in Cluster G1 and G4). Thus, compared with all the genes in the maize genome, scRNA-seq genes in Cluster G1 and G4 were differentially modified with histone marks.

Our analyses indicate that genes with distinctly prominent functions in leaf tissue, such as those in G1 and G4, are tempo–spatially regulated, at least partially, through epigenetic regulation during leaf development.

### 3.4. Construction of Transcription Factor (TF) Regulatory Network

**Figure 4:**
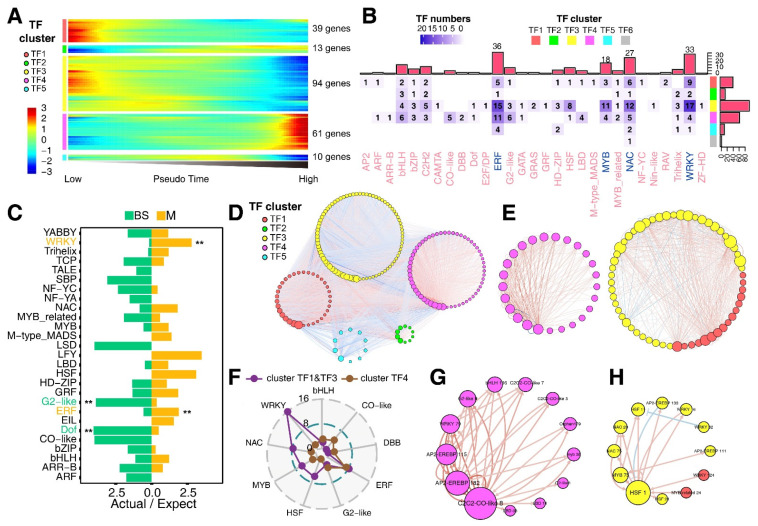
Identification of key transcription factors in M-cell development. (**A**) Heatmap showing the expression profiles of 217 highly dynamically expressed TFs ordered across pseudo-time in the 5 clusters. (**B**) Heatmap showing the number of TFs associated with different families in different gene clusters, the annotation bar plots on the top and the right of the heatmap show the numbers of TFs related to different families and clusters. (**C**) Bar plots showing the ratio of actual to expected numbers of TFs related to different families in M and BS cells. Significance test was determined for the ratios between M and BS cells (two-sided chi-squared test, ** indicates *p* < 0.01). (**D**) SCODE network displaying 191 dynamically expressed TFs with a cutoff as 0.1 during M-cell development. (**E**) SCODE network filtered with edge overlapping WGCNA network across M-cell development with a cutoff of 0.5, representing TFs involved in the early and the late stage of the leaf development. (**F**) Radar plot showing the TF numbers related to different families in Cluster TF1 and TF3 and Cluster TF4 network. (**G**) SCODE network filtered with edge overlapping WGCNA network across M-cell development with a cutoff of 0.5, representing TFs involved in the early stage of leaf development. (**H**) SCODE network filtered with edge overlapping WGCNA network across M-cell development with a cutoff of 0.5, representing TFs involved in the late stage of leaf development. The size of the node in all SCODE networks indicates the degree of the node.

Among a total of 217 TFs that showed dynamic pseudo-time expression profiles in transcription factor Cluster TF1-TF5 extracted from G1–G5, which represent the expression profiles of TFs in each cluster (Figure 4A), we found that *WRKY*, *ERF*, *NAC*, *MYB* and *HSF* TF families were dominant in Cluster TF1, TF3 and TF4 (Figure 4B). Genes in Cluster TF1 and TF3 were highly expressed in the low pseudo-time stage, whereas genes in Cluster TF4 were highly expressed in the high pseudo-time stage (Figure 4A). Thus, Cluster TF1/TF3 and TF4 genes might function in the early and late stage of M-cell development, respectively. To identify TF families preferentially expressed in BS and M cells, we compared ratios of the actual and expected numbers of highly expressed TFs separately in BS and M cells for each family using our newly generated bulk RNA-seq data (chi-squared test) (Figure 4C). Our analyses revealed that *WRKY* and *ERF* families were expressed at significantly higher levels in the M cells than those in the BS cells, which is in agreement with the previous findings in maize [6,7]. Moreover, *WRKY* and *ERF* families are also mesophyll cell-specific TFs in *Arabidopsis* [40]. Therefore, these findings indicate that both TF families may be essential in controlling M-cell development.

To further comprehend M-cell development in the maize leaf tissue, we constructed a gene regulatory network by using the linear Ordinary Differential Equations (ODE) algorithm for our scRNA-seq (SCODE) (see methods) [29] (Figure 4D). To confirm the accuracy of the edges in the network, we performed weighted gene coexpression network analysis (WGCNA) on the expressed genes identified in our scRNA-seq. All M cells were divided into 22 new cell clusters based on a higher-resolution parameter (Supplementary Table S4). Reads for each cell cluster were pooled to represent an independent M-cell sample using an average pseudo-time in the same cell cluster (Supplementary Table S4). We performed a WGCNA analysis using 22 pooled M-cell samples to obtain coexpression connections between genes pairs (Supplementary Table S5). Gene interactions overlapped between the SCODE and the coexpression network were retained for further constructing of condensed interaction networks. We obtained two small SCODE networks; one consisted of TF1 and TF3 TF genes, another had TF4 genes (Figure 4E). TF1- and TF3-dominant networks comprised *WRKY*, *ERF*, *NAC*, *MYB* and *HSF* families, which were highly expressed at the early stage during the development of M cells, whereas the TF4-dominant network mainly consisted of *ERF* family that were highly expressed at the late stage of the M-cell development (Figure 4F). Three *C2C2-CO-like* (*COL3*, *7* and *8*) TFs also played important roles in TF4-dominant network (Figure 4G), by contrast, *HSF* TFs were the core components in Cluster TF1- and TF3-dominant networks (Figure 4H). After comparing the pseudo-time trajectory of the published data, 21 of 25 (84%) key transcript factors from the networks of Cluster TF1, TF3 and TF4 showed the same expression profile (Supplementary Table S6). Consistently, the two hub genes from the online datasets, *HSF1* and *COL8*, displayed the same expression profiles across pseudo-time as our data (Supplementary Figure S4B).

### 3.5. Involvement of TF-Regulatory Network in the M-Cell Development

**Figure 5:**
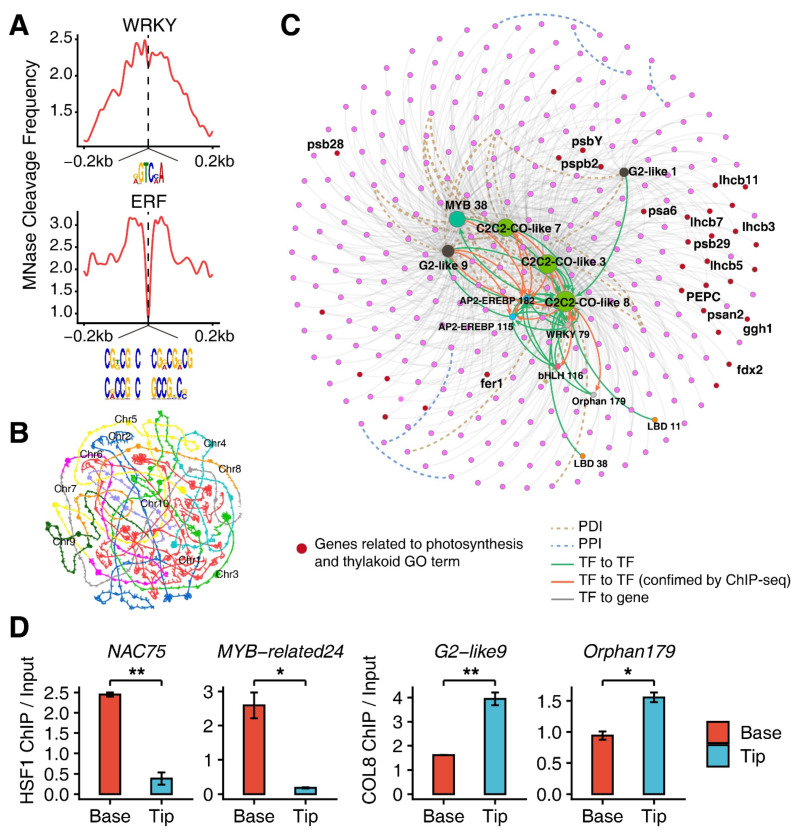
Construction of TF-regulatory network in M-cell development. (**A**) Footprints of WRKY and ERF TFs detected within the MNase-hypersensitive sites (MHSs), which were located within 500 bp of genes coexpressed with TFs. (**B**) Self-organized maps of chromatin interactions by H3K4me3-related loops. (**C**) The regulatory network for transcription factors and genes in Cluster 4. Green edges indicate predicted regulatory relationships between transcription factors. Orange edges indicate regulatory relationships between transcription factors confirmed by TF ChIP-seq. Grey edges indicate regulatory relationships between transcription factors and target genes. PDI and PPI mean promoter–distal interactions and promoter–promoter interactions generated from the chromatin interaction data, respectively. Genes related to photosynthesis are highlighted in the network. (**D**) ChIP-qPCR assay showing the differential binding signal of HSF1 and COL8 between the leaf bases and the leaf tips, *NAC75* and *MYB-related24* were targeted by HSF1 and *G2-like9* and *Orphan179* were targeted by COL8. The ChIP enrichment levels for *NAC75* and *MYB-related24* were presented as mean ± SD from two biological replicates, and for *G2-like9* and *Orphan179* were presented as mean ± SD from three technical replicates. Significance test was determined using one-sided *t*-test, * indicates *p* < 0.05, ** indicates *p* < 0.01.

To confirm whether these TFs regulate other genes in the same pseudo-time cluster, we identified MNase-hypersensitive sties (MHSs) located within 1 kb of G1, G3 and G4 cluster genes, and retrieved the corresponding DNA sequences. The MEME suite [41] was then employed to search the TF binding motifs stored in the C3C4 database (http://www.epigenome.cuhk.edu.hk/C3C4.html, accessed on 3 January 2022) in the retrieved DNA sequences. The majority of motifs in the C3C4 database displayed a high similarity with *Arabidopsis* motifs in the MEME database [42] (Supplementary Table S7). As anticipated, we indeed found that the binding motifs for *WRKY*, *NAC* and *MYB* families were enriched in the MHS peaks located within the promoters of 1838 genes in G1 and G3, and the motifs for *ERF* family were enriched in the MHS peaks within the promoters of 401 genes in G4 (Supplementary Table S7). The binding sites of WRKY and ERF were located at the centers of MNase cleavage regions (Figure 5A).

HiChIP data can be efficiently used to interrogate protein-centric spatial genomic locus interactions [43]. To generate TF-centric regulatory networks for the two groups of genes, we combined the published H3K4me3 HiChIP data [32] (Figure 5B) with 104 TF-related ChIP seq data [31]. We found that 1269 of 1838 G1 and G3 cluster genes were included in the regulatory network (Supplementary Figure S5). Notably, 17 genes for coding histones in the network, which was associated with nucleosome assembly and chromosome GO terms, were mostly regulated by *homeobox* (*HB*)*70*, and their counterparts *AtHB7* and *AtHB12* genes in *Arabidopsis* have a function in plant development [44]. The *HB70* gene was linked to 34 genes responsible for chloroplast development, which include *DXS* gene that encodes 1-deoxyxylulose 5-phosphate synthase activity essential for chloroplast development [45]. The *HB70* gene was also linked to the *PLL5* gene, which encodes phosphatase 2C-like protein and regulates leaf development since its knockout mutants have abnormally shaped leaves (Supplementary Table S8) [46]. The *HSFB1* gene of the *HSF1* genes in *Arabidopsis* play essential roles in programmed cell death (PCD) during plant development [47]. Moreover, 477 of 1269 (*ca.* 38%) genes encode various enzymes in the network. In addition, 318 of 401 genes in G4 were included in the regulatory network (Figure 5C). Among them, 32 genes associated with thylakoid and photosynthesis GO terms were highlighted in the network. *PEPC*, *ferritin1* (*fer1*) and *ferredoxin1* (*fdx2*) genes were directly regulated by COL8, of which the *PEPC gene* is a photosynthetic gene specifically expressed in M cells [6] and the *fer1* gene is located in plastids which are the source of iron for de novo synthesis of the cytochromes and FeS proteins during chloroplast development [48,49]. *Arabidopsis* plants with gene *fdx2* knockout exhibit lower growth rate and less chlorophyll [50], indicating the importance of this gene for plant development. All genes or TFs potentially involved in the base and tip differential developmental network are listed in Supplementary Table S8. In particular, we detected several spatial interactions between either WRKY or ERF and its corresponding coexpressed genes through analyzing the published H3K4me3 HiChIP data [32] (Supplementary Figure S6), indicating that the existence of tightly coupled TF-target pairs in stress response.

To further verify whether these TFs are indeed functionally involved in M-cell development, we further analyzed the published ChIP-seq data for 104 TFs [31]. An orthologous gene *Zm00001d012518* of *ipgam1/2* gene (2,3-biphosphoglycerate-independent phosphoglycerate mutase*1/2*) in maize displayed a declining trend in the expression levels across pseudo-time. The *ipgam 1/2* gene in rice functions in chlorophyll synthesis, photosynthesis, and chloroplast development [51], whilst its double mutants display reticulated leaves in *Arabidopsis* [52]. ChIP-seq peaks associated with the binding of MYB, ERF and WKRY TF families in G1 and G3 were found to be located directly in the promoter region of *Zm00001d012518* containing an MHS. (Supplementary Table S9). It has been documented that 62 genes with functional defects affect chloroplast biogenesis in maize, thereby reducing photosynthesis efficiency [15]. We found that 46 genes functioning in the chloroplast biogenesis were expressed in our scRNA data (Supplementary Figure S7), 17 genes were found to exhibit similar expression profiles to genes in G4 in Figure 2C [15]. ChIP-seq peaks associated with the binding of C2C2-CO and ERF TFs were found to be frequently present in the promoters of the 17 genes (Supplementary Table S9). Remarkably, *C2C2-COL8*, one of the hub genes in the SCODE network, was frequently associated with the 17 genes (Figure 4G and Supplementary Table S9). In addition, two target genes in the network for *HSF1* and *COL8* gene were selected separately for protoplast-based ChIP-qPCR assay, and *HSF1* gene showed an approximately 5.7- and 13.2-fold (base/tip) higher ChIP signal in the base within the promoters of *NAC75* and *MYB-related24*, respectively, while *COL8* gene was about 1.4- and 0.7-fold (tip/base) higher in the tip within the promoters of *G2-like9* and *Orphan179*, respectively. (Figure 5D).

Collectively, all above analyses show that two TF-centered regulatory networks differentially function in M-cell development.

### 3.6. Functions of HSF1 and COL8 Alone or Coordinated with Each Other in Regulating M-Cell Development

**Figure 6:**
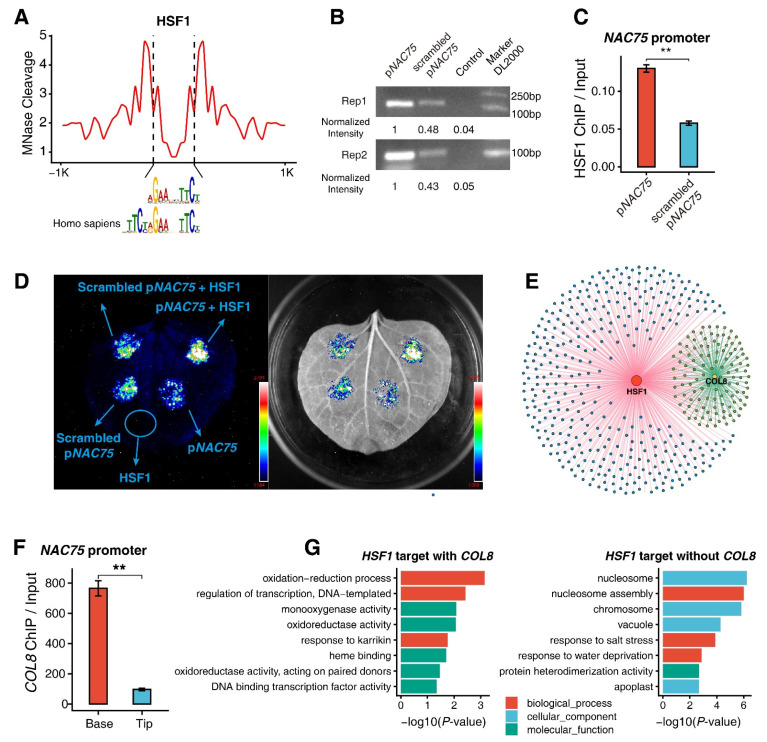
Validation of HSF1 binding motif. (**A**) Footprinting assay of the HSF1 binding motif. (**B**,**C**) HSF1 (**B**) IP-PCR or (**C**) IP-qPCR assays for assessing the binding of HSF1 with natural HSF1 binding sites and the corresponding scrambled sequences. (**D**) Dual-luciferase reporter assay using *Nicotiana benthamiana* leaves co-infiltrated with the combination of *P_NAC75_*: LUC with 35S::*HSF1*. (**E**) *HSF1*- and *COL8*-regulated network. (**F**) COL8 qPCR assays for assessing the binding of COL8 in *NAC75* promoter in leaf tip and base. (**G**) GO-term enrichment analyses for HSF1-targeted genes with or without the COL8 binding sites. The ChIP signal were presented as mean ± SD from three technical replicates in (**C**,**F**). Significance test was determined using one-sided *t*-test, ** indicates *p* < 0.01.

To assess how HSF1 regulates genes involved in the early development of M cells, we integrated our MH-seq with all 473 *HSF1*-coexpressed genes. We identified 429/473 (*ca.* 91%) genes with proximal MHSs. We also detected a clear MHS-related footprint corresponding to AGAAnnTTCT sites, which were identified from the *HSF1*-coexpressed genes with MHSs (Figure 6A). The motif is known as a conserved repetitive pattern of palindromic binding sequences for HSFs in the promoters of heat-shock-inducible genes of all eukaryotes [53,54]. Moreover, evidence from HSF1 IP-PCR (Figure 6B and Supplementary Figure S8) and dual-luciferase reporter gene assay (Figure 6C) further verified that HSF1 binds to the promoter of *NAC75* which contains a predictive HSF1 binding site (Figure 6D).

Interestingly, after searching MHS regions of *HSF1*-regulated genes in the network, we found that ±1 kb of 115 genes also contained COL8 ChIP-seq peaks (Figure 6D). The binding of COL8 to the promoter of *NAC75* in the leaf base was confirmed by the ChIP-qPCR assay (Figure 6E). These results indicated that some genes may be coregulated by HSF1 and COL8 in the early developmental stages during maize leave development. Thus, we classified the HSF1 binding genes into two subsets based on whether they were bound by COL8. After conducting GO-term enrichment analyses, we observed distinct GO terms for each subset of genes (Figure 6F). For example, the HSF1 binding genes with COL8 binding sites were mainly enriched with GO terms associated with oxidation–reduction processes and regulation of transcription or DNA binding activity (Figure 6F, left). By contrast, the HSF1 binding genes without COL8 binding were overrepresented in GO terms representing the biological processes of chromosomes and response to salt and water stresses (Figure 6F, right).

These analyses indicate *HSF1* and *COL8* may cooperate with each other in the leaf development in maize.

## 4. Discussion

### 4.1. Subfunctional Differentiation among Functionally Specialized M Cells

The maize leaf contains two distinct cell types with specialized functions, M and BS cells, to accommodate photosynthesis and metabolism [55]. M and BS cells are distinct in physical organization, gene expression, metabolic pathways and enzyme activity, which constitute an exquisite experimental system for interrogating the underlying mechanisms controlling leaf cell differentiation in relation to some functionalities. The regulatory mechanisms of functional differentiation between both cell types have been well-characterized at the transcriptional [6,7,56], biochemical [4,57] and epigenetic [12,58,59,60] levels in maize. In addition, developmental stage-related transcriptional and metabolic changes in maize leaves have also been studied [6,9]. However, the development of either a specialized M or BS cell is still under-researched, and is important to understanding chloroplast biogenesis, differentiation and functions. In addition, the bulk RNA-seq or other omic approaches in the previous studies primarily reflect the average levels of cell mixtures. It is almost impossible to distinguish cells from different development stages and correlate the development statuses of individual cells with functions. The advent of single-cell technology offers a powerful technique to characterize complex tissues or cell subtypes to understand their respective functions. In this study, due to failing to conduct BS single-cell RNA-seq, we mainly conducted M-cell-related scRNA-seq in combination with pseudo-time analyses, and identified six gene clusters based on the distinct gene expression profiles across them (Figure 2C), reflecting instances of gene subfunctionalization in M cells. In particular, our scRNA-seq showed at least two distinct gene subtypes (Cluster G1 and G4) with functional differentiation. Genes in Cluster G1, which were highly expressed in the early stage of leaf development, primarily functioned in some fundamental biological functions, whereas genes in Cluster G4, with an inverse expression profile compared to the genes in Cluster G1, peaked at the last stage of leaf development and primarily functioned in photosynthesis (Figure 2E). These two gene clusters are in agreement with previous findings about the developmental stages from the leaf base to the leaf tip [6,36]. scRNA-seq has been successfully applied to identify cell subpopulations, unveil regulatory relationships among genes, and track cell spatiotemporal developmental trajectories in human stem cells and cardiac progenitor cells [61,62] and *Arabidopsis* root tissue [63,64,65]. Our study provided evidence to show the development of M cells in the green leaf tissue from the single-cell level, a major place for photosynthesis and respiration, and some carbon assimilation pathways such as starch biosynthesis, nitrogen and sulfur assimilation. However, very few BS cells were obtained in our data, which is similar to the published data, indicating that capture of BS cells for scRNA-seq may not be easy. Comprehensive characterizations of M- and BS-cell-related scRNA-seq will provide more information about functional divergence between the two cell types in maize. Additionally, gene loss of function-related experimental validation needs to pinpoint functions of both gene clusters in leaf development in the future.

### 4.2. Key Transcriptional Factors in Regulation of M-Cell Development

Here, we found that some expression inconsistency of genes between Cluster G1 and G4 occurred between our scRNAs-seq and bulk RNA-seq (Figure 3A). The plausible explanations for the inconsistency are as follows: One is the sequencing methodology, as scRNA-seq technology barcoded each gene transcript in each single cell first, thus it only detected 9299 expressed genes for total 7234 single cells, with an average of 3500 expressed genes in each single cell. Among all 20,972 expressed genes identified by analyzing our bulk RNA-seq, the genes identified by analyzing our scRNA-seq primarily represented the top 17% of expressed genes in each single cell, thus the genes with medium and low expression levels in each individual single cell cannot be fully detected in scRNA-seq. In contrast, bulk RNA-seq pooled each RNA transcript from all cells followed by RNA-seq library preparation, and therefore, it could detect more gene transcripts, especially for those genes with medium and low expression levels; Another plausible explanation is that there was a diverse variation in the leaf material for the bulk RNA-seq compared to the protoplasts for scRNA-seq. Only 7254 single cells were analyzed in scRNA-seq, whereas approximately 5–10 M or even more cells were used for extracting total RNA for the bulk RNA-seq experiment.

Interactions between *trans*-acting factors and *cis*-acting elements are essential for the M- or BS-cell-type-related gene differential expression or differential accumulation of enzyme proteins for photosynthesis [13,59]. It is well-known that the M-cell-specific expression of *PEPC* gene is fine-tuned by interactions between the unique *cis*-acting elements located in promoter region and the DNA binding with one finger (DOF) 1 transcription factor, which is specifically expressed in M cells [66,67]. A few transcription factors, such as DOF, Maize Nuclear Factors (MNFs), PEP-I, SCARECROW (SCR) and SHORTROOT (SHR) have been proposed to be involved in differential gene expression between M and BS cells in maize or other C4 plants [9,11,59,68]. However, the involvement of transcription factors in subfunctional differentiation of M cells during maize leaf development is still inadequately characterized. Our study revealed that *WRKY* and *ERF* TF genes were preferentially expressed in M cells in the early and the late developmental stage of the maize leaf, respectively (Figure 4C). In addition, our study showed that a spatiotemporal regulation of differential leaf development was mediated by some key TFs through directly interacting with target genes or indirectly interacting with genes through coexpression networks. Furthermore, differential functions of HSF1 and COL8 TFs in the leaf base and tip were experimentally validated using ChIP-qPCR assay (Figure 5D). Interestingly, some of genes were coregulated by HSF1 and COL8 in the leaf base and tip, respectively, suggesting that the weak expression level of *COL8* in the base exhibits some biological relevance. Accumulating evidence shows that HSFs are required for plant/leaf development. HSFs can function in plant thermotolerance by regulating the expression of heat shock protein (HSP) genes, and in other abiotic stresses, such as dehydration, salinity, low temperature, H_2_O_2_, salicylic acid (SA) and abscisic acid (ABA) treatment, by regulating the expression of stress-responsive genes [69,70,71,72,73,74,75,76]. Overexpression of the *TaHsfA2-10* gene can increase content of chlorophyll in *Arabidopsis* [70], indicating possible involvement of the *HSF* gene in chloroplast development by activating *HSP* gene expression. *Hsp100/101* genes have been found to be essential for normal chloroplast development in *Arabidopsis* [77,78], while overexpression of chloroplast-targeted gene *Hsp101* homologue, *APG6*, resulted in pale-green leaves [78], indicating that constitutive overexpression of *APG6* cDNA had a negative effect on chloroplast biogenesis. Moreover, changes in *HsfB1* gene expression can reprogram metabolic pathways in the tomato leaf [79]. Of course, the above TF-regulated target genes and their contributions to photosynthesis and assimilation need further experimental validations using the defective mutants of TFs or their targets in the future.

By focusing on leaf single cells of various developmental stages, our study revealed some functionally distinct gene clusters with different expression patterns, and the key regulatory genes in M-cell development, thereby shedding some light on M-cell subfunctional differentiation. Our study showed that functional differentiation of M cells was largely spatiotemporally regulated at transcriptional levels with the involvement of epigenetic regulation and interactions.

## 5. Conclusions

Through utilization of scRNA-seq in combination with pseudo-time and gene regulatory network analyses, our study reveals *WRKY*, *ERF*, *NAC*, *MYB* and *HSF* TF families, especially *WRKY* and *ERF* families, are major determinants in the early and the late stages of maize M-cell development. TF regulatory network and Hi-C analyses further display occurrence of regulatory interactions between TFs and target genes. The interactions were validated using protoplast-based ChIP-qPCR assay, in particular, the binding site of HSF1 was experimentally confirmed. Thus, our study provides evidence showing how TFs function in M-cell development in maize.

## Data Availability

Accession numbers: The maize M-cell scRNA-seq (GSE138526), M- and BS-cells bulked RNA-seq data (GSE117982) and ChIP-seq data of H3K27me3, H3K4me3, H3K36me3 for M cells (GSE117964) were deposited in the NCBI Gene Expression Omnibus (GEO) (http://www.ncbi.nlm.nih.gov/geo/, accessed on 3 January 2022. Published ChIP-seq data of H3K27ac were obtained from SRA database (http://www.ncbi.nlm.nih.gov/sra, accessed on 3 January 2022) with accession number PRJNA391551. Published scRNA-seq data were obtained from GEO database with accession number GSE157759. Published ChIP-seq data of 104 transcript factors in maize were obtained from GEO database with accession number GSE137972.

## Supplementary Materials

The following supporting information can be downloaded at: https://www.mdpi.com/article/10.3390/genes13020374/s1, Dataset S1: The report of the single-cell RNA-seq data generated from the Cell Ranger software; Figure S1: Heatmap showing the correlation coefficient between our scRNA-seq and the published scRNA-seq data generated from maize seedlings; Figure S2: GO enrichment analysis of genes specifically expressed in Cluster C6 and C7; Figure S3: Pseudo-time trajectory of all M cells; Figure S4: Pseudo-time trajectory of the online maize scRNA-seq data; Figure S5: Regulatory network for transcript factors and genes in Cluster 1 and 3; Figure S6: H3K4me3 HiChIP network showing genes connected by chromatin loop for TFs; Figure S7: Expression heatmap exhibiting 46 genes required for chloroplast biogenesis across the pseudo-time; Figure S8: Schematic workflow of HSF1-IP-PCR assay; Table S1: Summary of primer information for qPCR assay; Table S2: Specifically expressed marker genes for cell cluster C6 and C7 generated using FindAllMarkers function in seurat package; Table S3: Pseudo-time expression trend of maize genes, whose orthologous genes in *Arabidopsis* function in the leaf development; Table S4: Summary of sequencing information for the 22 subclusters of M cells; Table S5: Edges of WGCNA network generated from the 22 pooled M-cell samples (Weight > 0.2); Table S6: Pseudo-time trajectory of key transcription factors from the regulatory network in the published data; Table S7: Motif identification for TFs overrepresented in the M cells; Table S8: List of genes or TFs potentially involved in the leaf base and the leaf tip network; Table S9: Summary of the potential binding of TFs in genes as indicated using ChIP-seq assay.
