# Supplementary material for: Single-Cell Transcriptome and Network Analyses Unveil Key Transcription Factors Regulating Mesophyll Cell Development in Maize
Source: Genes (Basel). 2022 Feb 20;13(2):374. doi: 10.3390/genes13020374 (PMC8872562; doi:10.3390/genes13020374)
Supplement: Supplementary file 1 [file genes-13-00374-s001.zip › Dataset S1 Cell Ranger Report.html]

MaizeB73-M-cell — Cell Ranger
 


Cell Ranger · MaizeB73-M-cell ·

{{ alerts.length}}

SUMMARY
    
ANALYSIS

The analysis detected some serious issues.  
 Details »

| Alert | | Value | Detail |
| --- | --- | --- | --- |
|  | **{{ alert.title }}** | {{ alert.value }} | {{ alert.message }} |

# Estimated Number of Cells

14,656

## Mean Reads per Cell

44,805

## Median Genes per Cell

2,148

Number of Reads
:   Total number of read pairs that were assigned to this library in demultiplexing.

Valid Barcodes
:   Fraction of reads with barcodes that match the whitelist after barcode correction.

Sequencing Saturation
:   The fraction of reads originating from an already-observed UMI. This is a function of library complexity and sequencing depth. More specifically, this is the fraction of confidently mapped, valid cell-barcode, valid UMI reads that had a non-unique (cell-barcode, UMI, gene). This metric was called "cDNA PCR Duplication" in versions of Cell Ranger prior to 1.2.

Q30 Bases in Barcode
:   Fraction of cell barcode bases with Q-score >= 30, excluding very low quality/no-call (Q <= 2) bases from the denominator.

Q30 Bases in RNA Read
:   Fraction of RNA read bases with Q-score >= 30, excluding very low quality/no-call (Q <= 2) bases from the denominator. This is Read 1 for the Single Cell 3' v1 chemistry and Read 2 for the Single Cell 3' v2 chemistry.

Q30 Bases in Sample Index
:   Fraction of sample index bases with Q-score >= 30, excluding very low quality/no-call (Q <= 2) bases from the denominator.

Q30 Bases in UMI
:   Fraction of UMI bases with Q-score >= 30, excluding very low quality/no-call (Q <= 2) bases from the denominator.

# Sequencing

|  |  |
| --- | --- |
| Number of Reads | 656,666,388 |
| Valid Barcodes | 96.6% |
| Sequencing Saturation | 47.4% |
| Q30 Bases in Barcode | 95.7% |
| Q30 Bases in RNA Read | 93.1% |
| Q30 Bases in Sample Index | 94.4% |
| Q30 Bases in UMI | 95.1% |

Reads Mapped to Genome
:   Fraction of reads that mapped to the genome.

Reads Mapped Confidently to Genome
:   Fraction of reads that mapped uniquely to the genome. If a gene mapped to exonic loci from a single gene and also to non-exonic loci, it is considered uniquely mapped to one of the exonic loci.

Reads Mapped Confidently to Intergenic Regions
:   Fraction of reads that mapped uniquely to an intergenic region of the genome.

Reads Mapped Confidently to Intronic Regions
:   Fraction of reads that mapped uniquely to an intronic region of the genome.

Reads Mapped Confidently to Exonic Regions
:   Fraction of reads that mapped uniquely to an exonic region of the genome.

Reads Mapped Confidently to Transcriptome
:   Fraction of reads that mapped to a unique gene in the transcriptome. The read must be consistent with annotated splice junctions. These reads are considered for UMI counting.

Reads Mapped Antisense to Gene
:   Fraction of reads confidently mapped to the transcriptome, but on the opposite strand of their annotated gene. A read is counted as antisense if it has any alignments that are consistent with an exon of a transcript but antisense to it, and has no sense alignments.

# Mapping

|  |  |
| --- | --- |
| Reads Mapped to Genome | 93.7% |
| Reads Mapped Confidently to Genome | 83.2% |
| Reads Mapped Confidently to Intergenic Regions | 12.5% |
| Reads Mapped Confidently to Intronic Regions | 0.9% |
| Reads Mapped Confidently to Exonic Regions | 69.8% |
| Reads Mapped Confidently to Transcriptome | 59.9% |
| Reads Mapped Antisense to Gene | 0.5% |

Barcode Rank Plot
:   {{ chart.description }}

Estimated Number of Cells
:   The total number of barcodes associated with cell-containing partitions, estimated from the barcode count distribution.

Fraction Reads in Cells
:   The fraction of valid-barcode, confidently-mapped-to-transcriptome reads with cell-associated barcodes.

Mean Reads per Cell
:   The total number of sequenced reads divided by the number of barcodes associated with cell-containing partitions.

Median Genes per Cell
:   The median number of genes detected per cell-associated barcode. Detection is defined as the presence of at least 1 UMI count.

Total Genes Detected
:   The number of genes with at least one UMI count in any cell.

Median UMI Counts per Cell
:   The median number of UMI counts per cell-associated barcode.

# Cells

|  |  |
| --- | --- |
| Estimated Number of Cells | 14,656 |
| Fraction Reads in Cells | 94.4% |
| Mean Reads per Cell | 44,805 |
| Median Genes per Cell | 2,148 |
| Total Genes Detected | 28,765 |
| Median UMI Counts per Cell | 10,075 |


# Sample

|  |  |
| --- | --- |
| Name | MaizeB73-M-cell |
| Description |  |
| Transcriptome | B73V4 |
| Chemistry | Single Cell 3' v3 |
| Cell Ranger Version | 3.0.2 |

{{ filter.selected }}   

- {{ value }}

#### {{ title }}:

{{ chart.description }}

## {{ chart.title }}

{{ chart.description }}

## {{ chart.title }}

{{ chart.description }}

## {{ chart.title }}

|  |  |  |  |  |
| --- | --- | --- | --- | --- |
|  |  | Cluster {{ $index/2 }} | |  |
| {{ col.label }} |
| {{ cell }} | {{ cell.f }} |

{{ chart.description }}

## {{ chart.title }}

{{ chart.description }}

## {{ chart.title }}
