# Supplementary material for: Single-Cell Transcriptome and Network Analyses Unveil Key Transcription Factors Regulating Mesophyll Cell Development in Maize
Source: Genes (Basel). 2022 Feb 20;13(2):374. doi: 10.3390/genes13020374 (PMC8872562; doi:10.3390/genes13020374)
Supplement: Supplementary file 1 [file genes-13-00374-s001.zip › Supplementary Figures.pdf]

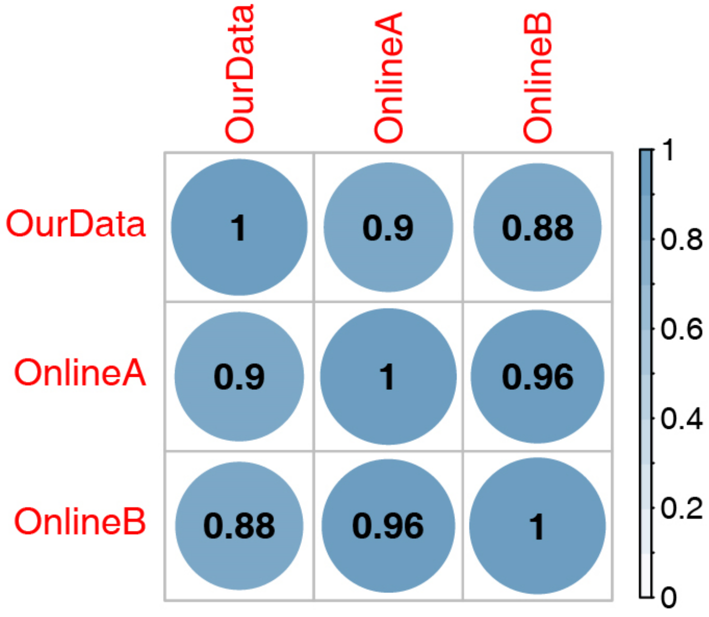

Supplementary Figure S1. Heatmap showing the correlation coefficient between our scRNA-seq and the published scRNA-seq data generated from maize seedlings.

The correlation coefficient was calculated using Spearman correlation test.

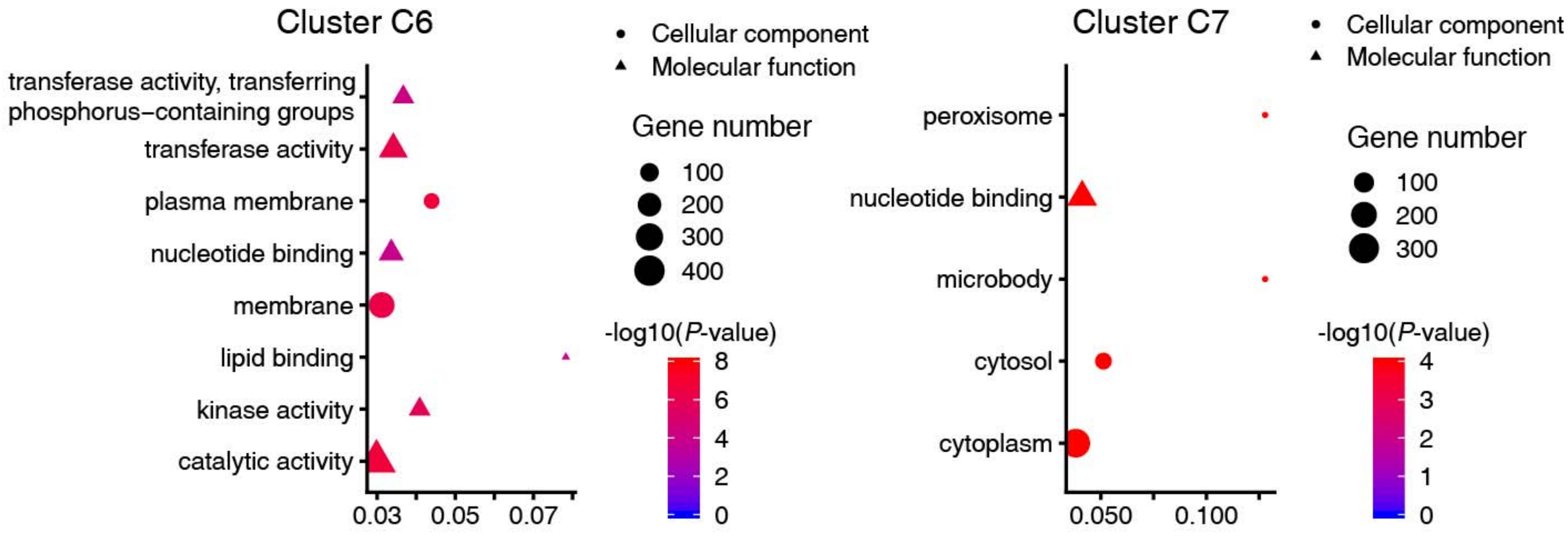

Supplementary Figure S2. GO enrichment analysis of genes specifically expressed in Cluster C6 and C7. Dotplot showing the GO terms of genes specifically expressed in Cluster C6 and C7. The *x* axis represents the number of genes in the list /the number of genes in the background. The color of each dot represents -$\log_{10}^{(P\text{-value})}$, the size of each dot represents the number of genes in the list, and the dot shape represents the GO term category.

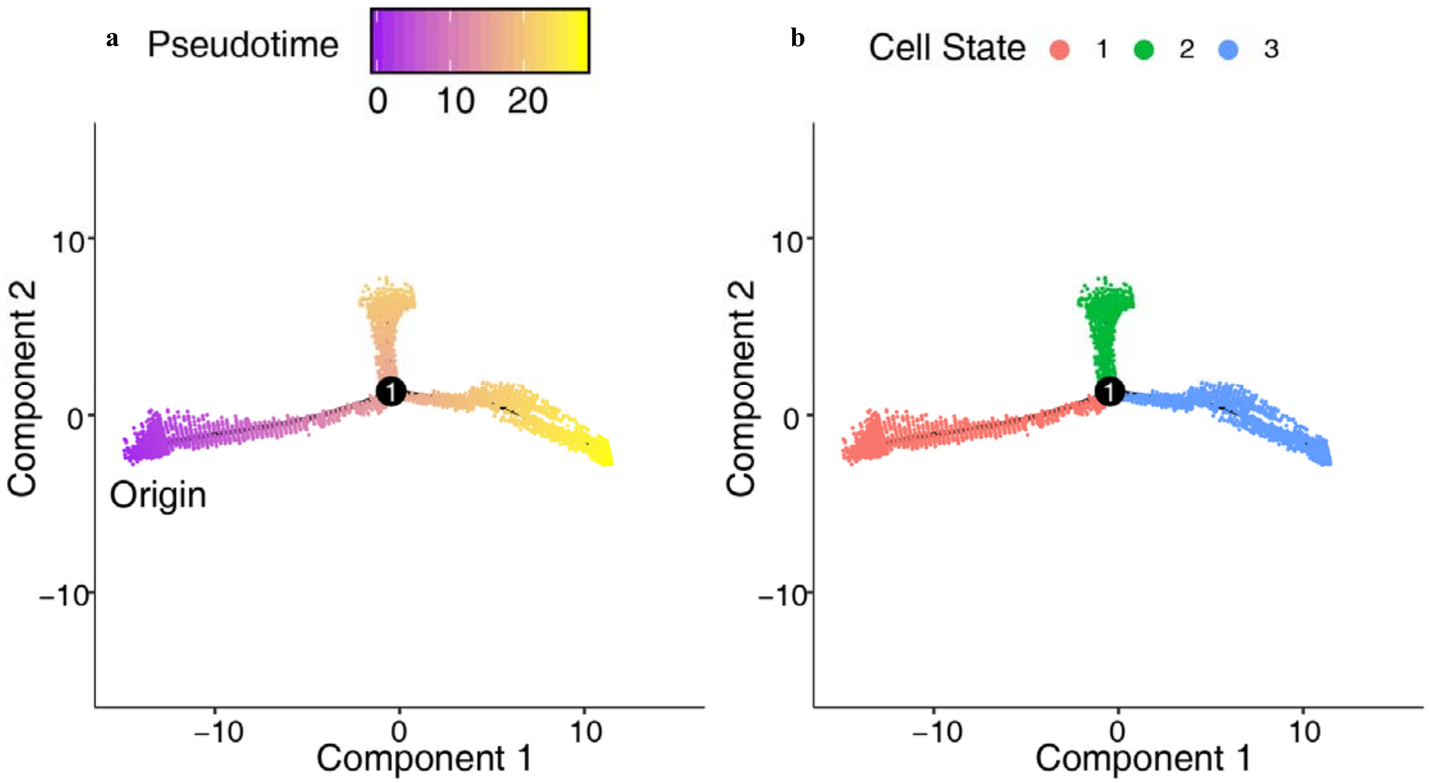

Supplementary Figure S3. Pseudo-time trajectory of all M cells. A The scatter plot illustrating the Pseudo-time trajectory. Each point represents a single M cell; the color of the cell represents the pseudo-time of the cell. The text "Origin" represents the defined root of the trajectory. **B** The scatter plot illustrating Pseudo-time trajectory of cell states. Each point represents a single M cell; the color of the cell represents a different cell state generated by monocles. The root can be switched to each end of the states.

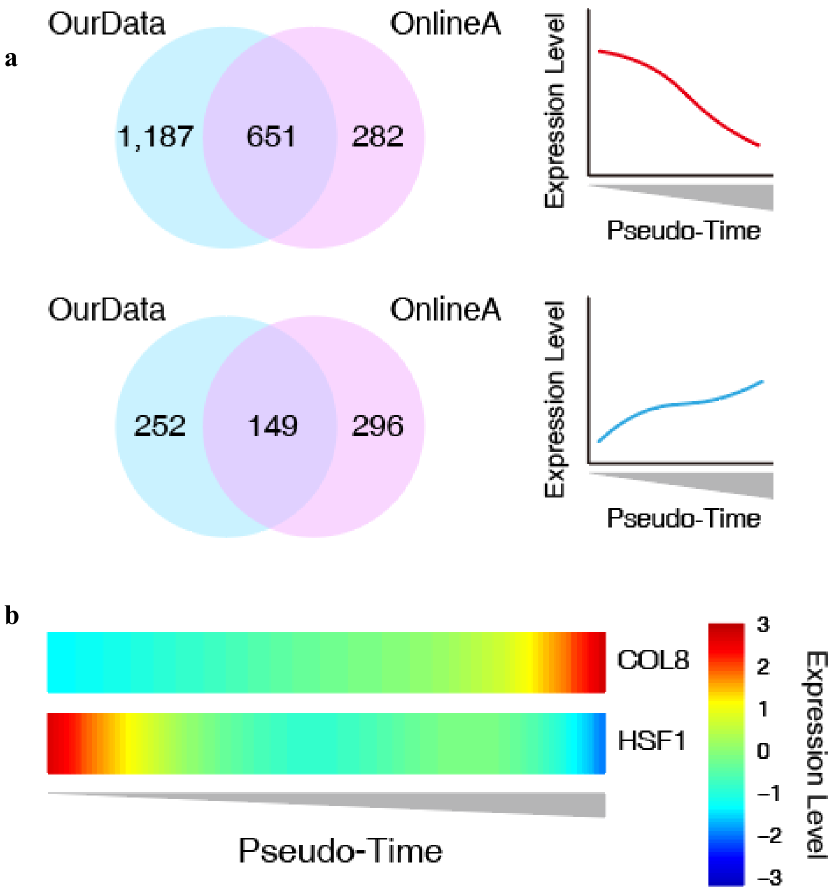

Supplementary Figure S4. Pseudo-time trajectory of the online maize scRNA-seq data. A Venn diagram showing the intersections of DEGs across pseudo-time between our data and the published scRNA-seq datasets (Bezrutczyk *et al.*, 2021). The top panel indicates genes highly expressed in the lower pseudo-time while the bottom panel indicates genes highly expressed in the higher pseudo-time. **B** Expression profiles of COL8 and HSF1 in the published scRNA-seq data set (Bezrutczyk *et al.*, 2021).

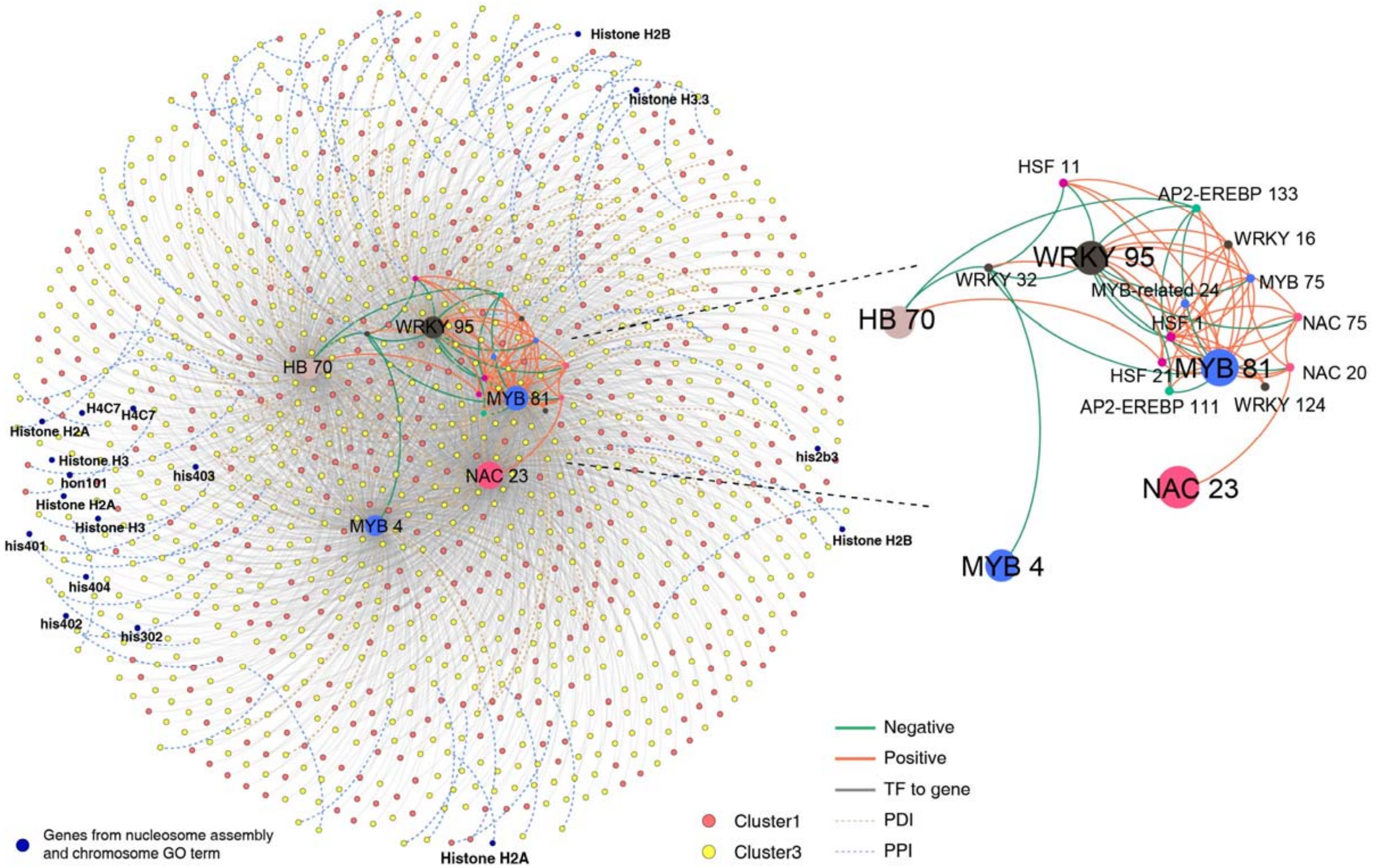

Supplementary Figure S5. Regulatory network for transcript factors and genes in Cluster 1 & 3. The zoomed panel showing the regulatory relationship between transcript factors. Green and orange edges between transcript factors represent predicted negative and positive regulatory relationships. Grey edges represents regulatory relationship between transcript factors and target genes. PDI and PPI mean promoter-distal interaction and promoter-promoter interaction generated from the chromatin interaction data, respectively.

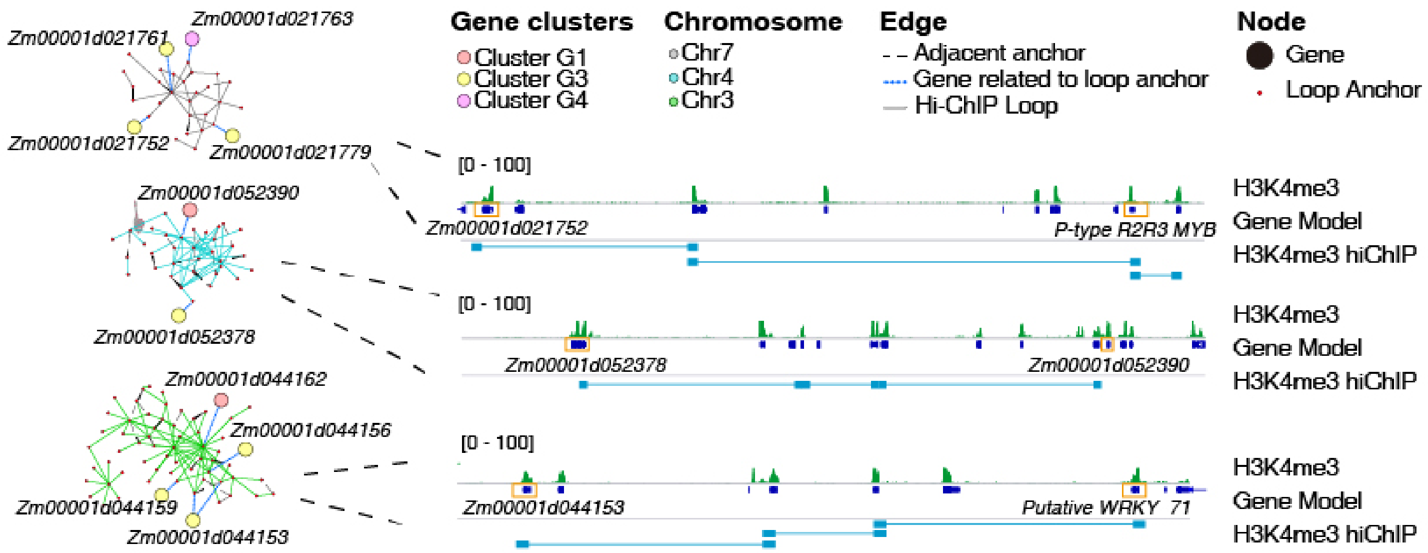

Supplementary Figure S6. H3K4me3 HiChIP network showing genes connected by chromatin loop for TFs. Bigger dots represent genes associated with the loop anchor region; small dots represent loop anchors in a 5 kb region. Solid lines represent connections of two anchors by H3K4me3 Hi-ChIP loops. Dashed lines represent adjacent anchor connections. Arrows represent connections between loop anchors and genes. IGV snap shots showing co-expressed genes and TFs connected by H3K4me3 HiChIP loops.

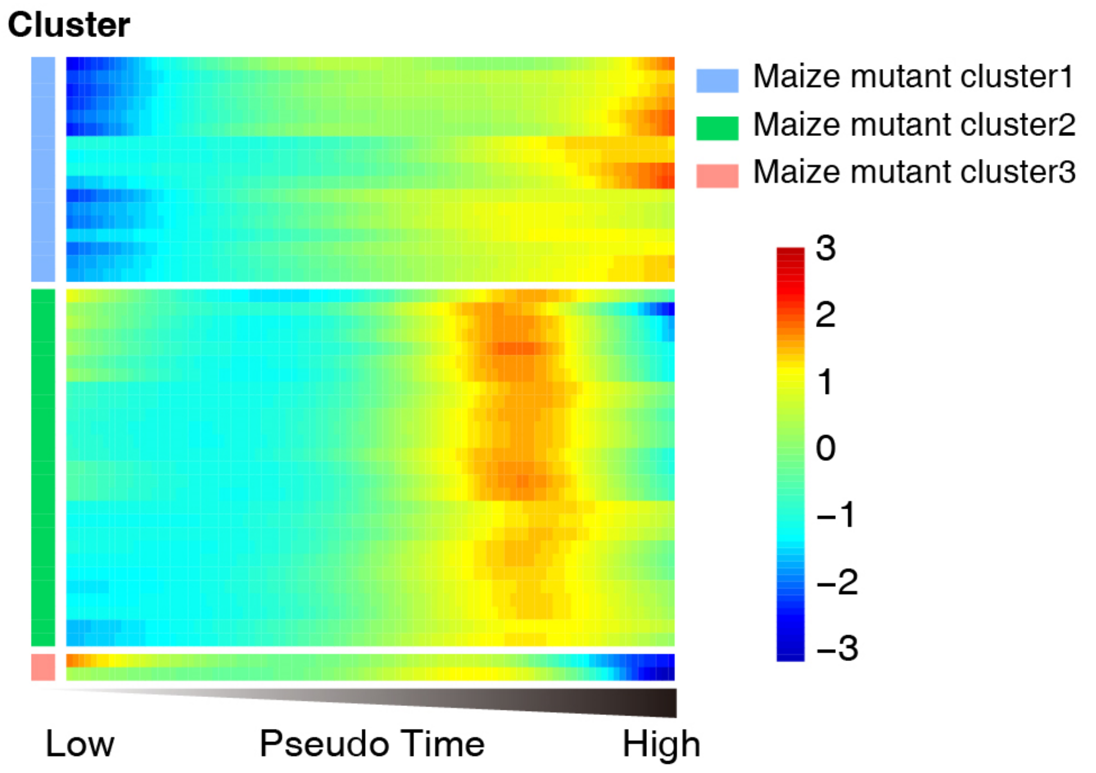

Supplementary Figure S7. Expression heatmap exhibiting 46 genes required for chloroplast biogenesis across the pseudo-time. It has been documented that functional defects in these genes affect photosynthesis (Belcher *et al.*, 2015).

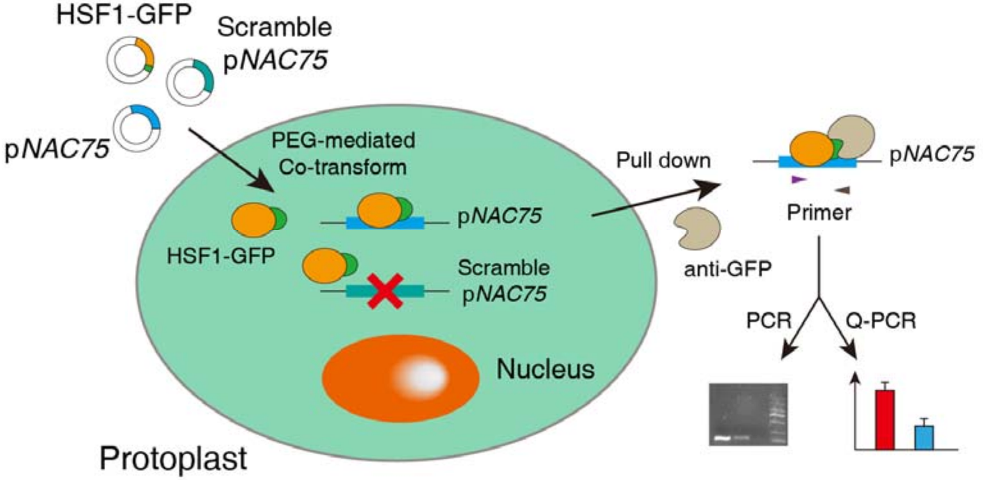

Supplementary Figure S8. Schematic workflow of HSF1-IP-PCR assay. HSF1 overexpressed plasmid DNA and plasmid containing promoter of *NAC75 (PNAC75)* with HSF1 binding and corresponding scrambled sequences were co-transformed into M cells. Anti-GFP antibody was used to pull down the GFP fused with HSF1.
